# Opioid use disorders and hospital palliative care among patients with gastrointestinal cancers: Ten-year trend and associated factors in the U.S. from 2005 to 2014

**DOI:** 10.1097/MD.0000000000020723

**Published:** 2020-06-19

**Authors:** Jinwook Hwang, Jay J. Shen, Sun Jung Kim, Sung-Youn Chun, Pearl C. Kim, Se Won Lee, David Byun, Ji Won Yoo

**Affiliations:** a Department of Thoracic and Cardiovascular Surgery, Korea University Medical Center, Ansan Hospital, Korea University College of Medicine, Ansan, South Korea; b Department of Health Care Administration and Policy School of Public Health, University of Nevada Las Vegas, Las Vegas, Nevada; c Department of Health Administration and Management, Soonchunhyang University, Asan, South Korea; d Department of Physical Medicine and Rehabilitation, Mountain View Hospital, Las Vegas, Nevada; e Department of Internal Medicine, Southern Nevada Veterans Affairs Health System, North Las Vegas, Nevada; f Department of Internal Medicine, University of Nevada Las Vegas, School of Medicine, Las Vegas, Nevada.

**Keywords:** cannabis use disorders, gastrointestinal neoplasms, hospital charges, in-hospital mortality, opioid use disorders, palliative care

## Abstract

This study aimed to analyze the trends of opioid use disorders, cannabis use disorders, and palliative care among hospitalized patients with gastrointestinal cancer and to identify their associated factors.

We analyzed the National Inpatient Sample data from 2005 to 2014 and included hospitalized patients with gastrointestinal cancers. The trends of hospital palliative care and opioid or cannabis use disorders were analyzed using the compound annual growth rates (CAGR) with Rao-Scott correction for *χ*^2^ tests. Multivariate logistic regression analyses were performed to identify the associated factors.

From 2005 to 2014, among 4,364,416 hospitalizations of patients with gastrointestinal cancer, the average annual rates of opioid and cannabis use disorders were 0.4% (n = 19,520), and 0.3% (n = 13,009), respectively. The utilization rate of hospital palliative care was 6.2% (n = 268,742). They all sharply increased for 10 years (CAGR = 9.61%, 22.2%, and 21.51%, respectively). The patients with a cannabis use disorder were over 4 times more likely to have an opioid use disorder (Odds ratios, OR = 4.029; *P* < .001). Hospital palliative care was associated with higher opioid use disorder rates, higher in-hospital mortality, shorter length of hospital stay, and lower hospital charges. (OR = 1.527, 9.980, B = −0.054 and −0.386; each of *P* < .001)

The temporal trends of opioid use disorders and hospital palliative care use among patients with gastrointestinal cancer increased from 2005 to 2014, which is mostly attributed to patients with a higher risk of in-hospital mortality. Cannabis use disorders were associated with opioid use disorders. Palliative care was associated with both reduced lengths of stay and hospital charge.

## Abstract 2

Supplemental Digital Content is available in the text

## 1 Introduction

Every year over 1.7 million people are diagnosed with cancer in the United States.^[1]^ Most of the patients suffered from cancer pain and controllable symptoms during their end-of-life time. For that reason, the integration of palliative care into standard oncology care regardless of the severity of the cancer is insisted upon in current oncology treatment.^[2]^

Opioid medication can mitigate the cancer-related symptoms and provide comfort to patients along with the cancer treatment. Palliative care guideline suggests the use of opioid drugs for cancer patients.^[3]^

There have been growing concerns about the possibility of opioid misuse and overuse, particularly in patients who suffered from chronic pain, such as cancer pain or chronic low back pain.^[4,5]^ For gastrointestinal cancer patients, weak opioids such as codeine, to strong opioid medications, such as morphine, oxycodone, hydromorphone, or fentanyl, could be prescribed to control pain and relieve anxiety.^[6]^

In 2016, more than 11.5 million people reported misuse of prescription pain medicine,^[7]^ and 115 Americans die every day from an opioid overdose.^[8]^ On October 27, 2017, the government administration declared the opioid crisis a national public health emergency under section 319 of the Public Health Service Act. This declaration was renewed on October 18, 2018, because of the continued consequences of the opioid epidemic.

Cannabis is classified as a Schedule I controlled substance because of its high potential for dependence and uncertain medical benefits. Over the past 2 decades, citizen-initiated public votes have driven medical and recreational cannabis use. Thirty three states and the District of Columbia have adopted marijuana legalization in some form (National Conference of State Legislatures, 2018). Cannabis remains the most commonly used illicit drug in the United States, with an estimated 22.2 million people using it currently, and an additional 2.4 million people reporting first-time use annually.^[9]^ As a consequence, there were increasing emergency department visits related to cannabis use from 2006 to 2014.^[10]^ Although there is an ongoing debate about whether cannabis is a gateway drug^[11]^ or a substitute for opioid use,^[12]^ little attention has been given to the impacts of marijuana legalization on opioid-related hospitalization among patients with gastrointestinal cancer.

This study aimed to investigate the trends of opioid use disorders, cannabis use disorders, and hospital palliative care among hospitalized patients with gastrointestinal cancer and to identify their associated factors.

## 2 Methods

### 2.1 Data source

This study was based on the Nationwide Inpatient Sample (NIS). NIS is the largest publicly available, all-payer U.S. hospital inpatient dataset. It contains a 20% stratified sample of hospital inpatient stays from across the U.S. The dataset captures discharge information from hospital inpatient stays and belongs to the family of the Healthcare Cost and Utilization Project (HCUP) sponsored by the Agency for Healthcare Research and Quality (AHRQ).^[13]^ The NIS can be weighted to generate national estimates. We used a 10-year data from 2005 to 2014. The use of the NIS dataset is entirely anonymous, with no risk of a confidentiality breach. An Institutional Review Board approval was not required. We completed a data user agreement with the AHRQ before using the NIS database.

### 2.2 Patient cohort selection

The 2005 to 2014 NIS datasets were used for analysis. We applied survey weights and adjustments to provide annual national population estimates. Our population of interest was hospitalized patients with gastrointestinal cancer. Patients were identified as having cancer in the digestive tract, including oral, esophagus, stomach, liver, gall bladder, pancreas, small intestine, and large intestine as the principal diagnosis, opioid use (abuse, dependence, unspecified use, poisoning), and palliative care by using the International Classification of Diseases, Ninth Revision, Clinical Modification (ICD-9-CM) codes (Supplementary Table 1). Quality control procedures performed by the HCUP have demonstrated reliability and accuracy, mainly when data contains the principal diagnosis.^[14]^ Patient-level characteristics from the database included age, gender, race, number of comorbidities (as a continuous variable), the severity of illness using all-patient refined diagnosis-related group (APR-DRG), the primary payer (Medicare, private insurance, Medicaid, others), and the zip code-based annual median household income.

### 2.3 Variables of interest

The primary outcomes were the proportion of opioid use disorders, cannabis use disorder, palliative care, and in-hospital death. We calculated the total cost of each hospitalization provided by HCUP by applying hospital-specific and group-average all-payer inpatient cost-to-charge ratios from the Centers for Medicare and Medicaid Services (CMS) to the reported hospital charges. Hospital charges were then adjusted for the annual inflation rate using CMS estimates and were expressed as annual means with 95% confidence intervals (C.I.s) in 2014 U.S. dollars.^[15]^

### 2.4 Statistical analysis

First, the compound annual growth rate (CAGR) was used to quantify temporal trends of the annual number of opioid use disorders and cannabis use disorders, and hospital palliative care in patients with gastrointestinal cancer. Its statistical significance was tested by Rao-Scott correction for *χ*^2^ tests for categorical variables. The CAGR supposes that year A is x and year B is y, and CAGR = (y/x) ^[1/(B-A)]-1^ has been widely used for health care valuation.^[16,17]^ Multivariate logistic regression analyses were performed to determine the relationship of patient demographics and socioeconomic status with cannabis use disorders, opioid use disorders, hospital palliative care, in-hospital mortality, length of hospital stay (LOS), and hospital charges.

To evaluate the impact of missing data on opioid use disorders, we compared the baseline characteristics between the missing and analyzed samples characteristics. We calculated imputed absent hospital charges using regression analysis models. There were no statistical differences between the baseline characteristics of the interpreted and missing data. The model was determined to be stable, and the assumption of randomly missing data was found to be reasonable using the observed data. All analyses were performed using SAS statistical software version 9.4 (SAS Institute Inc., Cary, NC). All reported *P* values were 2-tailed, and *P* value <.05 was considered statistically significant.

## 3 Results

### 3.1 Descriptive data and temporary trends of hospitalization of patients with gastrointestinal cancer

From 2005 to 2014, the NIS database contained 77,394,755 hospital inpatient stays and 4,364,416 hospitalizations of patients with gastrointestinal cancer. Table 1 presents the descriptive characteristics of patients and hospitalizations. Opioid use disorders and cannabis use disorder were less than 1.0% of the total hospitalizations (0.4%, n = 19,520; 0.3%, n = 13,009); 6.2% of the patients received palliative care during the hospitalization (n = 268,742). Overall in-hospital mortality rate was 6.7% (n = 294,567). The average LOS and inflation-adjusted hospital charges were 7.3 days and $43,077 per hospitalization, respectively.

**Table 1:**
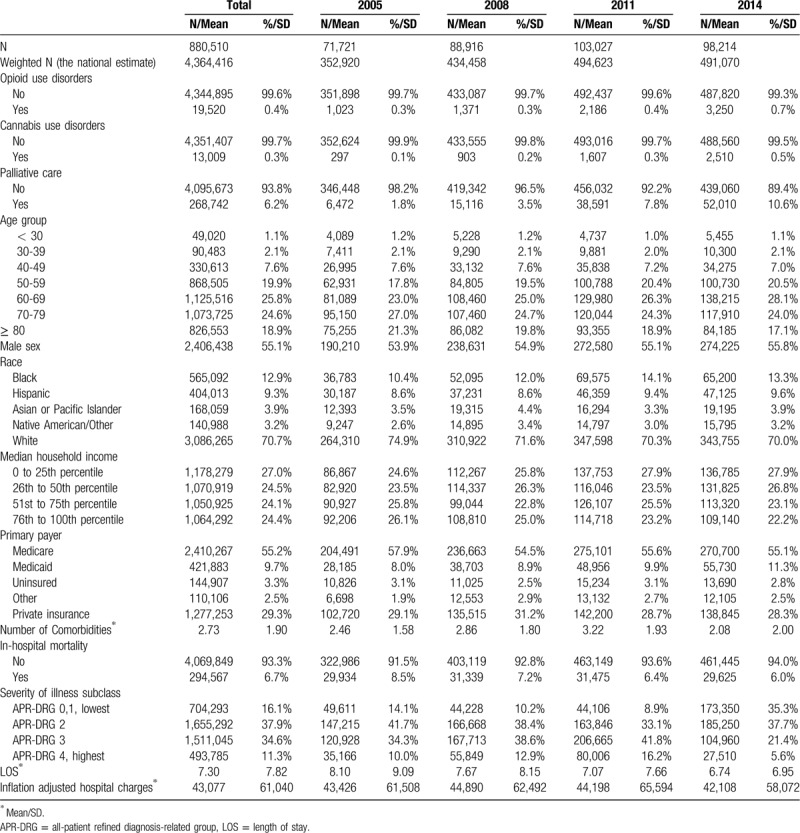
Characteristics of the population (N = 4,364,416).

**Figure 1:**
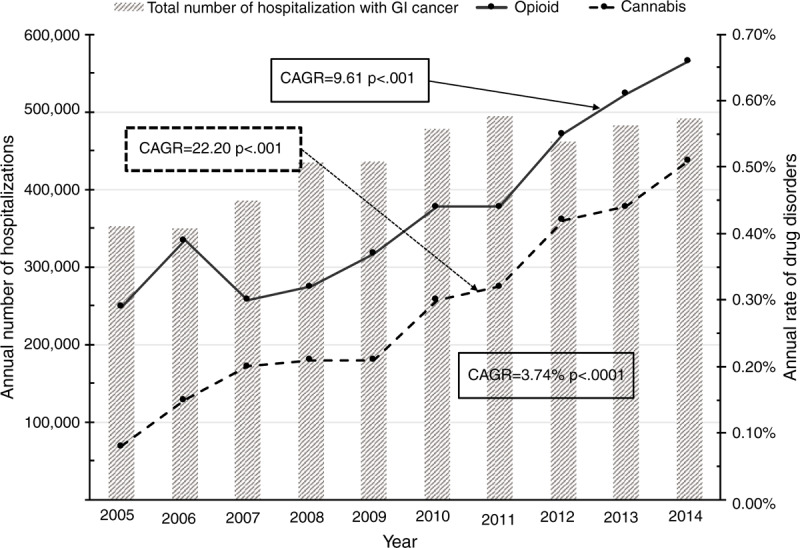
The temporal trends of the total number of hospitalizations with gastrointestinal cancers and opioid, and cannabis-use disorders among them from 2005 to 2014. ∗CAGRs: The compound annual growth rates.

Figure 1 presents the trends and the CAGRs of opioid use disorders, cannabis use disorders, and the annual numbers of hospitalization. The annual rate of opioid use disorders, cannabis use disorder, and hospitalization have increased over the past decade (CAGR = 9.61%, 22.20%, and 3.74%; each *P* < .0001).

**Figure 2:**
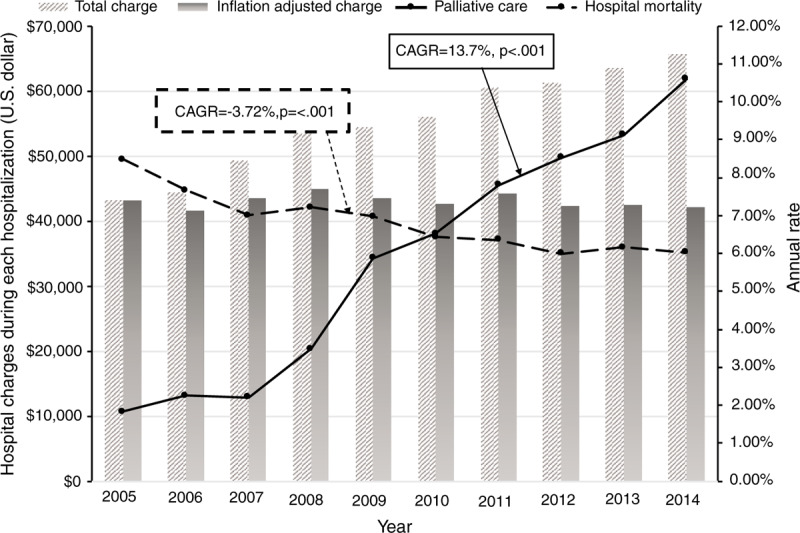
The temporal trends of total hospital charges, inflation-adjusted hospital charges during each hospitalization, and the annual rate of hospital palliative care and mortality among patients with gastrointestinal cancers from 2005 to 2014. ^∗^CAGRs: The compound annual growth rates.

Hospital palliative care has increased by over 5 times from 2005 (1.8%) to 2014 (10.6%) (Table 1). There was a sharp increase in the period from 2008 to 2009 (Fig. 1). In-hospital mortality, LOS, and inflation-adjusted hospital charges have decreased over time (CAGR = −3.72%, −2.02% and −0.34%); each of *P* < .0001) (Table 1 and Fig. 2).

### 3.2 Factors associated with opioid use disorders, palliative care, length of hospital stay, and hospital charges (log-transformed multivariate regression analysis)

Table 2 shows the factors associated with opioid use disorders. The patients with a cannabis use disorder were more likely related to opioid use disorders by over 4 times than patients without it (Odds ratio, OR = 4.029, 95% Confidence Interval, CI = 3.367–4.823). Patients who received palliative care during the hospitalization were more associated with opioid use disorders (OR = 1.423, 95%CI = 1.263–1.604). Younger groups, Black and Hispanic races, and public health insurance beneficiaries were more vulnerable to opioid use disorders (see Table 2).

**Table 2:**
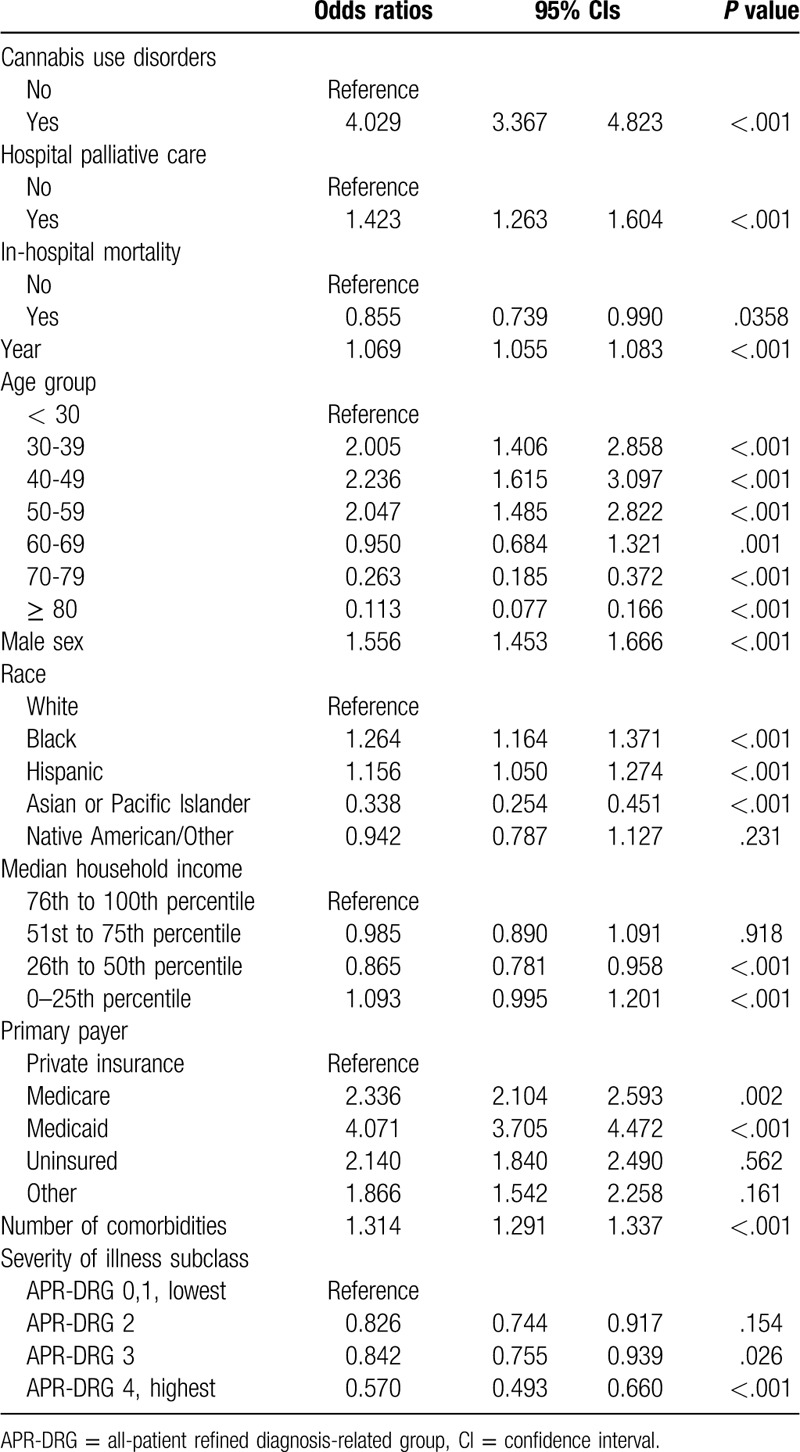
Factors associated with opioid use disorders.

Table 3 presents the odds ratios of hospital palliative care. Patients of older ages were more likely to receive palliative care consultation or services. Hospital palliative care was associated with an increase in in-hospital mortality (OR = 9.980, 95%CI = 9.734–10.233). Opioid and cannabis use disorders were not related to the rise of in-hospital mortality (Supplementary Table 2). Private insurance beneficiaries and uninsured patients showed a higher tendency of in-hospital mortality than the public insurance beneficiaries and others.

**Table 3:**
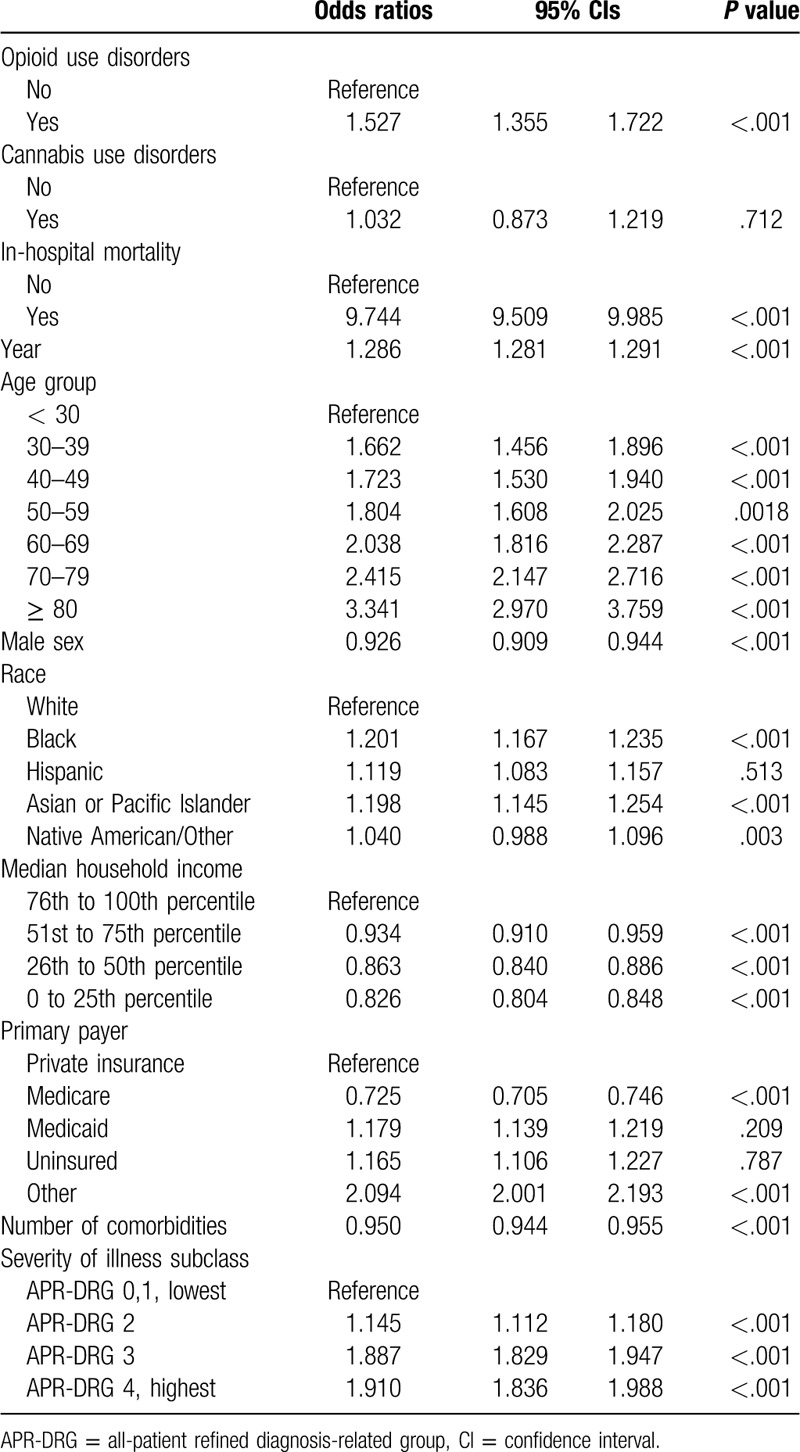
Factors associated with hospital palliative care.

Tables 4 and 5 shows the factors associated with the LOS and hospital charges (inflation-adjusted). Palliative care was associated with reductions in the length of hospital stay and hospital charges. Opioid or cannabis use disorders were not associated with an increase in the LOS and hospital charges.

**Table 4:**
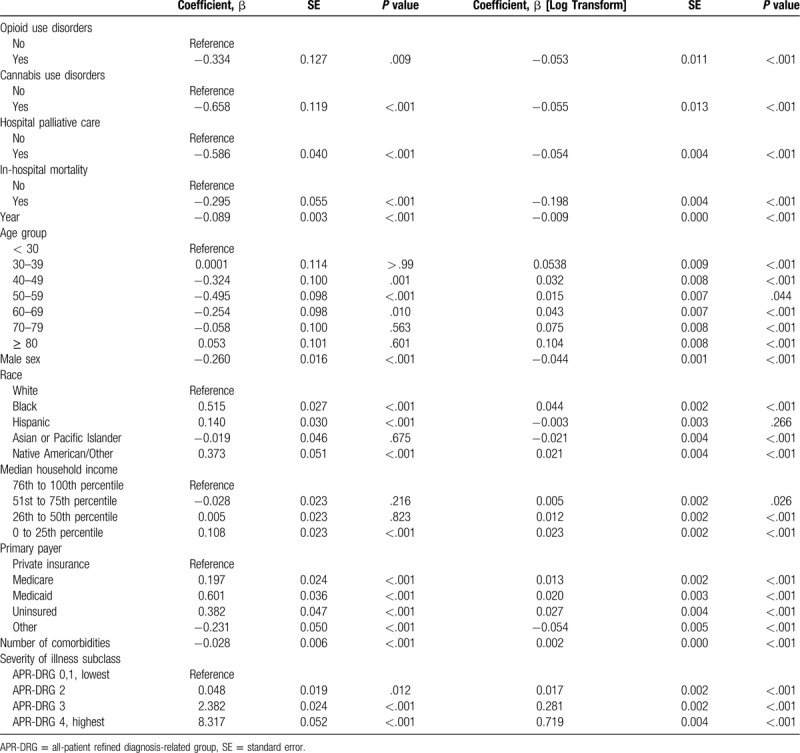
Factors associated with the length of hospital stay.

**Table 5:**
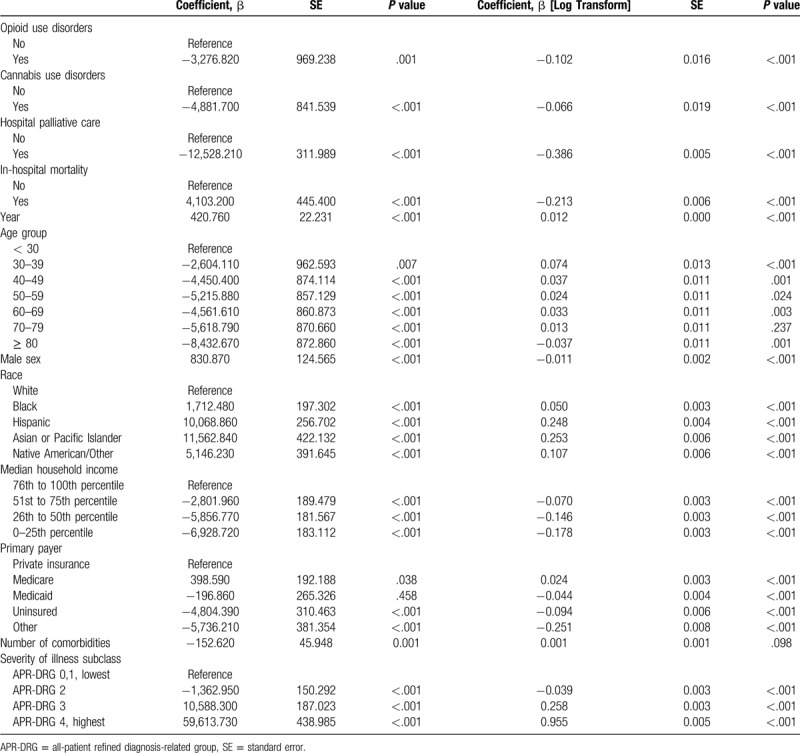
Factors associated with hospital charges (Inflation-adjusted).

## 4 Discussion

### 4.1 The trends and associated factors of opioid use disorders in patients with gastrointestinal cancer

The trend indicated an increase in opioid use disorders among hospitalizations of patients with gastrointestinal cancer (see Table 1 and Fig. 1). It is consistent with the nationwide trend of an opioid epidemic.^[18]^ The overuse and misuse of prescribed opioids were the leading causes of this issue, and both patients and their caregivers and family were affected. We could observe an incremental trend of cannabis use disorders, as well (Table 1). A recent study suggested that medical cannabis law appears to have contributed to an increasing prevalence of illicit cannabis use and cannabis use disorders.^[19]^

In the regression analysis to investigate the association of cannabis use disorders and opioid use disorders, patients who were diagnosed with a cannabis use disorder were over 4 times more likely to have an opioid use disorder (Table 2). From the early 90s, in the west coast states, such as California, Oregon, and Washington, medical cannabis legalization has been enacted to reduce opioid overuse death. The law has appeared to be valid.^[20]^ However, increasing cannabis use disorder rates cause a growing concern about opioid and other substance use disorders.^[9]^ Cannabis use disorder rates increase along with the increasing trend of opioid use disorders in our analysis. On October 19, 2009, the government issued a memo stating that it would not prosecute marijuana users and sellers who complied with the state laws for cannabis use.^[21]^ We speculated that the year 2010 would be the beginning of an opioid epidemic phase because of the drug policies influencing public attitudes and behaviors on opioids, cannabis, and other substances. Although there is an ongoing debate whether marijuana is a gateway drug, our findings imply that cannabis and opioid use can mutually increase each other, based on the reports from the survey^[22]^ and claim data.^[10]^ Continuous use of cannabis for either medicinal or recreational purposes may lead to increased dependence and higher tolerance levels. Therefore, the medical and recreational use of marijuana can potentially lead to more severe health outcomes, such as cannabis abuse or addiction. Although the prevalence of cannabis use disorders is still lower in hospitalized patients, the medical and illicit use of cannabis to younger patients need to be monitored to prevent further substance addiction as with the Prescription Drug Monitoring Programs for opioid overdose. Because younger age is at higher risk of cannabis use disorders than opioid use disorders.

### 4.2 The underutilization, disparities, and late referral to hospital palliative care in patients with gastrointestinal cancer

Our study demonstrates that the hospital palliative care for gastrointestinal cancer has increased considerably over the past 10 years (Fig. 2). This remarkable increase was mostly due to the accumulating evidence of palliative care benefits, an increase in the number of hospital palliative care programs, and implementation of the Affordable Care Act.^[23,24]^

However, only 6.16% of the patients could be served by palliative care for 10 years. Palliative cares were underutilized for hospitalized patients even in cases of advanced cancer.^[24]^ In the real world, palliative care was commonly utilized by patients who were dying.^[23]^ As an extension of the hospital palliative care, some reports show us the probability of in-home palliative care.^[25]^ Systematic implementation of ambulatory and hospital palliative services need to be extended among the broader population.^[26]^

We could notice the delayed referral to palliative care (Table 3). The patients who were severely sick and of older age (Medicare) were referred for hospital palliative care, resulting in palliative care being associated with higher odds of in-hospital mortality (Table 3). In the study of the pattern of palliative care delivery using the SEER-Medicare linked database, 40% of the patients received palliative care consults in the last 7 days of life.^[27,28]^ Promotion of earlier provision of palliative care consultation is needed at the time of enrollment to the cancer registry.^[29,30]^

### 4.3 Safety of palliative care related to opioid use disorders

In our study, we observed that in-hospital mortality and the length of hospital stay decreased over the past 10 years, contrary to the increase in the total number of hospitalizations (see Fig. 1). It could be interpreted in that more patients with gastrointestinal cancers choose their home or community rather than hospitals as the place for their end-of-life (EOL) time. A recent study reported that the terminally ill patients tend to spend their EOL time to stay with their family or caregivers out of the hospital, rather than in the intensive care unit.^[31]^ Palliative care highlights the advanced care plan. If widely utilized, palliative care, and advanced care planning can help to encourage the patient decision where they stay and whom they stay with at their EOL.^[32]^ A broader distribution of home or community base palliative and hospice care is needed to meet this trend for this population as well.^[33]^

In our study, opioid and cannabis use disorders did not increase the in-hospital mortality rates (Supplementary Table 2). However, the opioid use disorder rates were associated with the hospital palliative care (Table 3). The American society of clinical oncology recommended the Prescription Drug Monitoring Programs (PDMPs) for pain medication of patients with cancer, and opioid-induced death of these patients are 10 times less likely than those of the general population.^[34–36]^ In our study, it is difficult to recognize whether it comes from prescribed medicinal or non-medical purpose consumption because the NIS data set shows all diagnoses at discharge. No matter what the association between opioid use disorder and palliative care is, our result implies that physicians or palliative care specialists should pay attention to check the history of prescription of opioids and diagnoses of opioid use disorders.

### 4.4 Effectiveness of hospital palliative care related to in-hospital mortality, length of hospital stay, and hospital charges

Hospital charges for patients with gastrointestinal cancers slightly decreased from 2005 to 2014 (CAGR = −0.34%, *P* < .0001) (Fig. 2). Most previous studies have reported that early introduction of palliative care shows favorable effects on health care utilization and costs, such as decreased LOS, in-hospital death, and hospital cost.^[37,38]^ In a recent meta-analysis from 6 observational studies about the economics of hospital palliative care, May et al^[38]^ reported that early hospital palliative care within 3 days of admission reduced the hospital cost for patients with cancer by $4,251 compared to general care. The cost-saving from reduced LOS and reduced intensity of treatment has been estimated to accrue due to early palliative care.

### 4.5 Limitations and strengths

Since this study was a retrospective review of hospital discharge-based data, there are several significant limitations. First, this study only included discharge data, and no actual medical assessments were included. In addition, the hospital discharge-based database does not provide information on actual consumption, dosage, or use patterns of opioids, cannabis, or other substances. Second, we relied on the ICD-9-CM codes that may have limited accuracy in capturing the actual number of persons who used opioids, cannabis, and other substances with or without mental health conditions because of incorrect coding or missing data from coding practices and awareness of clinicians differences.^[10]^ Furthermore, the identification of persons who used illicit drugs with mental health conditions was significantly underestimated, considering the low sensitivity and high specificity in the weighted estimates from the discharge dataset.^[39]^ This study cannot address the potential for unrecognized coding errors or unreported events that could influence the results. Third, our analysis could not fully specify the stages and onsets of gastrointestinal cancers. Besides, the temporal relationship, that is, the opioid use disorders occurred before admission that was the cause of hospitalization, or occurred during the hospitalization, as an unintended overdose from the hospital-prescribed medication was not investigated. We were also not able to examine whether cannabis use was initiated before the opioid use. Future studies need to consider this information using other datasets. Given the significant and recent data from a nationally representative dataset, we believe that the temporal trends and associated factors of opioid use disorders among hospitalized patients with gastrointestinal cancers are likely generalizable to most patients with gastrointestinal cancers.

## 5 Conclusion

This study shows that opioid use disorders and hospital palliative care among hospitalizations of patients with gastrointestinal cancers steadily increased from 2005 to 2014 in U.S. hospitals. Opioid use disorders were associated with cannabis use disorders and palliative care. Prescription of opioids or dispensing cannabis for patients with cancers should be performed with caution. Delayed provision of palliative care could be associated with higher in-hospital mortality. Even though the overall rate of provision of palliative care was still low, the hospital palliative care for patients with gastrointestinal cancers showed a cost-saving effect.

## Acknowledgments

We acknowledge all of the HCUP Data Partners that contribute to HCUP. A current list of the HCUP Data Partners is linked the following the HCUP-US web page (www.hcup-us.ahrq.gov/hcupdatapartners.jsp).

## Author contributions

**Conceptualization:** Pearl C. Kim, Ji Won Yoo.

**Data curation:** Jay J. Shen.

**Formal analysis:** Jay J. Shen, Sun Jung Kim, Sung-Youn Chun.

**Investigation:** Jay J. Shen, Sun Jung Kim, Pearl C. Kim, Ji Won Yoo.

**Methodology:** Jay J. Shen, Sung-Youn Chun.

**Project administration:** Pearl C. Kim, David Byun.

**Resources:** Ji Won Yoo.

**Software:** Sun Jung Kim.

**Supervision:** Jay J. Shen, Se Won Lee.

**Validation:** Sung-Youn Chun, Ji Won Yoo.

**Visualization:** Jinwook Hwang.

**Writing – original draft:** Jinwook Hwang.

**Writing – review & editing:** Jinwook Hwang, Jay J. Shen, Se Won Lee.

## Supplementary Material

Supplemental Digital Content

## Supplementary Material

Supplemental Digital Content
