# Supplementary material for: Opioid use disorders and hospital palliative care among patients with gastrointestinal cancers: Ten-year trend and associated factors in the U.S. from 2005 to 2014
Source: Medicine (Baltimore). 2020 Jun 19;99(25):e20723. doi: 10.1097/MD.0000000000020723 (PMC7310906; doi:10.1097/MD.0000000000020723)
Supplement: Supplemental Digital Content [file medi-99-e20723-s001.docx]

**Supplementary Table 1. ICD-9-CM codes used for gastrointestinal cancer, substance use, and palliative care**

| Principal diagnosis | Descriptions | ICD-9-CM codes |
| --- | --- | --- |
| Malignant neoplasm of the GI tract | Oral, esophagus, stomach, colon, rectum | 1498,1499,1500,1501,1502,1503,1504,1505,1508,1509,1510,1512,1513,1514,1515,1516,1518,1519,1520,1521,1522,1523,1528,1529,1530,1531,1532,1533,1534,1535,1536,1537,1538,1539,1540,1541,1542,1543,1548 |
|  | Liver, pancreas, gallbladder, peritoneum | 1550,1551,1552,1560,1561,1562,1568,1569,1570,1571,1572,1573,1574,1578,1579,1580,1588,1589,1590,1591,1598,1599 |
| Substance use (abuse, dependence, unspecified use, poisoning) | Opioid | 304.00, 304.01, 304.02, 304.03, 304.70, 304.71, 304.72, 304.73, 305.50, 305.51, 305.52, 305.53, 965.00, 965.01, 965.02, 965.09,.0, E850.0, E850.1, E850.2, E850.2, E850.9, E935.0, E935.1, E935.2 |
|  | Alcohol | 303.00, 303.01, 303.02, 303.03, 303.90, 303.91, 303.92, 303.93, 305.00, 305.01, 305.02, 305.03, 980.0, 980.1, 980.2, 980.3, 980.8, 980.9 |
|  | Marijuana | 304.30, 304.31, 304.33, 305.20, 305.21, 305.22, 305.23 |
|  | Sedative | 304.10, 304.11, 304.13, 305.40, 305.41, 305.42, 305.43, 967.0, 967.1, 967.2, 967.3, 967.4, 967.5, 967.6, 967.8, 967.9, 969.4, E851, E852.0, E852.1, E852.2, E852.3, E852.4, E852.5, E852.8, E852.9, , E853.0, E853.1, E853.2, E853.8, E853.9, E937.0, E937.1, E937.2, E937.3, E937.4, E937.4, E937.5, E937.6, E937.7, E937.8, E937.9 |
|  | Cocaine | 304.20, 304.22, 304.23, 305.60, 305.61, 305.62, 305.63, 970.81, 970.89 |
|  | Stimulant | 304.00, 304.41, 304.42, 304.43, 305.70, 305.71, 305.72, 305.73, 969.6, 970.0, 970.1, 970.9, E854.2, E854.3, E854.9 |
|  | Hallucinogen | 304.50, 304.51, 304.52, 304.53, 305.30, 305.31, 305.32, 305.53, E854.1, E855.5, E855.6, E855.8, E855.9 |
|  | Other substances | 304.60, 304.61, 304.62, 304.63, 304.70, 304.71, 304.72, 304.73, 304.80, 304.81, 304.83, 304.90, 304.91, 304.92, 304.93, 305.80, 305.81, 305.82, 305.83, 305.90, 305.91, 305.92, 305.93, 969.0, E939.0, E939.1, E939.2, E939.2, E939.3, E939.4, E939.5, E939.6, E939.6, E939.7, E939.8, E939.9 |
| Palliative care | | V66.7 |

Notes: ICD-9, International Classification of Diseases, 9^th^ revision; GI, gastrointestinal
