# Supplementary material for: Opioid use disorders and hospital palliative care among patients with gastrointestinal cancers: Ten-year trend and associated factors in the U.S. from 2005 to 2014
Source: Medicine (Baltimore). 2020 Jun 19;99(25):e20723. doi: 10.1097/MD.0000000000020723 (PMC7310906; doi:10.1097/MD.0000000000020723)
Supplement: Supplemental Digital Content [file medi-99-e20723-s002.docx]

| **Supplementary Table 2. Factors associated with in-hospital mortality** | | | | |
| --- | --- | --- | --- | --- |
|  | **Odds Ratios** | **95% CIs** | | **P-Value** |
| **Opioid use disorders** |  |  |  |  |
| No | Reference |  |  |  |
| Yes | 0.871 | 0.748 | 1.014 | .074 |
| **Cannabis use disorders** |  |  |  |  |
| No | Reference |  |  |  |
| Yes | 0.507 | 0.392 | 0.655 | <.001 |
| **Hospital palliative care** |  |  |  |  |
| No | Reference |  |  |  |
| Yes | 9.980 | 9.734 | 10.233 | <.001 |
| **Year** | 0.923 | 0.920 | 0.926 | <.001 |
| **Age group (years)** |  |  |  |  |
| < 30 | Reference |  |  |  |
| 30-39 | 1.403 | 1.206 | 1.632 | <.001 |
| 40-49 | 1.798 | 1.572 | 2.057 | <.001 |
| 50-59 | 2.222 | 1.949 | 2.532 | <.001 |
| 60-69 | 2.566 | 2.251 | 2.924 | <.001 |
| 70-79 | 3.156 | 2.765 | 3.601 | <.001 |
| ≥ 80 | 3.701 | 3.241 | 4.226 | <.001 |
| **Male sex** | 1.225 | 1.202 | 1.249 | <.001 |
| **Race** |  |  |  |  |
| White | Reference |  |  |  |
| Black | 1.126 | 1.095 | 1.159 | .0103 |
| Hispanic | 1.033 | 1.000 | 1.068 | <.001 |
| Asian or Pacific Islander | 1.173 | 1.121 | 1.227 | <.001 |
| Native American/Other | 1.126 | 1.070 | 1.185 | .1228 |
| **Median household income** |  |  |  |  |
| 76th to 100th percentile | Reference |  |  |  |
| 51st to 75th percentile | 0.992 | 0.965 | 1.019 | <.001 |
| 26th to 50th percentile | 1.056 | 1.028 | 1.085 | .7337 |
| 0 to 25th percentile | 1.175 | 1.144 | 1.206 | <.001 |
| **Primary payer** |  |  |  |  |
| Private insurance | Reference |  |  |  |
| Medicare | 0.809 | 0.787 | 0.831 | <.001 |
| Medicaid | 0.984 | 0.949 | 1.021 | <.001 |
| Uninsured | 1.185 | 1.125 | 1.248 | <.001 |
| Other | 1.483 | 1.410 | 1.559 | <.001 |
| **Number of comorbidities** | 0.909 | 0.903 | 0.914 | <.001 |
| **Severity of illness subclass** |  |  |  |  |
| APR-DRG 0,1, lowest | Reference |  |  |  |
| APR-DRG 2 | 0.863 | 0.834 | 0.892 | <.001 |
| APR-DRG 3 | 1.686 | 1.631 | 1.742 | <.001 |
| APR-DRG 4, highest | 9.424 | 9.098 | 9.761 | <.001 |

CI, confidence interval; APR-DRG, All-Patient Refined Diagnosis-Related Group
